# Performance Evaluation of Image Segmentation Using Dual-Energy Spectral CT Images with Deep Learning Image Reconstruction: A Phantom Study

**DOI:** 10.3390/tomography11050051

**Published:** 2025-04-27

**Authors:** Haoyan Li, Zhenpeng Chen, Shuaiyi Gao, Jiaqi Hu, Zhihao Yang, Yun Peng, Jihang Sun

**Affiliations:** 1 Department of Radiology, Beijing Children’s Hospital, Capital Medical University, National Center for Children’s Health, No. 56, Nanlishi Road, Xicheng District, Beijing 100045, China; lihaoyanxx111@126.com (H.L.); gsy_19951114@outlook.com (S.G.); jiaqih2024@163.com (J.H.); 18614268596@163.com (Z.Y.); 2 Qingdao Academy of Chinese Medical Sciences, Shandong University of Traditional Chinese Medicine, Qingdao 266112, China; 18366108084@163.com; 3 Children’s Hospital of Xinjiang Uygur Autonomous Region, Xinjiang Hospital of Beijing Children’ s Hospital, Urumqi 830054, China

**Keywords:** multi-slice CT, dual-energy CT, image enhancement, U-Net model, deep learning, image reconstruction

## Abstract

**Objectives**: To evaluate the medical image segmentation performance of monochromatic images in various energy levels. **Methods**: The low-density module (25 mm in diameter, 6 Hounsfield Unit (HU) in density difference from background) from the ACR464 phantom was scanned at both 10 mGy and 5 mGy dose levels. Virtual monoenergetic images (VMIs) at different energy levels of 40, 50, 60, 68, 74, and 100 keV were generated. The images at 10 mGy reconstructed with 50% adaptive statistical iterative reconstruction veo (ASIR-V50%) were used to train an image segmentation model based on U-Net. The evaluation set used 5 mGy VMIs reconstructed with various reconstruction algorithms: FBP, ASIR-V50%, ASIR-V100%, deep learning image reconstruction (DLIR) with low (DLIR-L), medium (DLIR-M), and high (DLIR-H) strength levels. U-Net was employed as a tool to compare algorithm performance. Image noise and segmentation metrics, such as the DICE coefficient, intersection over union (IOU), sensitivity, and Hausdorff distance, were calculated to assess both image quality and segmentation performance. **Results**: DLIR-M and DLIR-H consistently achieved lower image noise and better segmentation performance, with the highest results observed at 60 keV, and DLIR-H had the lowest image noise across all energy levels. The performance metrics, including IOU, DICE, and sensitivity, were ranked in descending order with energy levels of 60 keV, 68 keV, 50 keV, 74 keV, 40 keV, and 100 keV. Specifically, at 60 keV, the average IOU values for each reconstruction method were 0.60 for FBP, 0.67 for ASIR-V50%, 0.68 for ASIR-V100%, 0.72 for DLIR-L, 0.75 for DLIR-M, and 0.75 for DLIR-H. The average DICE values were 0.75, 0.80, 0.82, 0.83, 0.85, and 0.86. The sensitivity values were 0.93, 0.91, 0.96, 0.95, 0.98, and 0.98. **Conclusions**: For low-density, non-enhancing objects under a low dose, the 60 keV VMIs performed better in automatic segmentation. DLIR-M and DLIR-H algorithms delivered the best results, whereas DLIR-H provided the lowest image noise and highest sensitivity.

## 1. Introduction

Automatic segmentation technology for medical images is widely used in clinical diagnosis, lesion measurement, radiotherapy, and other medical fields and plays a crucial role in accurately segmenting, measuring lesions, and planning radiation therapy. The quality of the CT image used for segmentation affects the results of automatic segmentation [1,2,3,4,5,6,7,8]. Dual-energy spectral computed tomography (DEsCT) offers expanded diagnostic capabilities by providing virtual monochromatic images (VMIs) of various photon energy levels, such as low kilo-electron volt (keV) images, to enhance the detection of small iodine contrast-enhancing lesions and improve image contrast compared to traditional CT [9,10,11,12,13]. This improved contrast of low-keV images in contrast-enhanced DEsCT can also be converted to reduce the radiation dose or contrast agent usage. In general, image noise increases as the energy level decreases in DEsCT, and iterative reconstruction (IR) algorithms such as the adaptive statistical iterative reconstruction veo (ASIR-V) have been used to reduce image noise. However, research indicates that the use of high-strength IR algorithms may lead to alterations in image texture, and the image texture change, together with the different image noises and CT values at various energy levels, could potentially impact the efficiency of artificial intelligence (AI) in automatic segmentation. This influence extends to the broader application of AI in CT imaging, where both efficiency and accuracy are critical [6,14,15,16]. Recently, a deep learning image reconstruction (DLIR) algorithm (TrueFidelity^TM^, GE HealthCare, Milwaukee, WI, USA) was introduced and incorporated into DEsCT and has demonstrated its ability to significantly reduce image noise without significantly altering the image texture compared to IR algorithms, resulting in improved image quality even under reduced radiation dose conditions [17,18]. Integrating DLIR with spectral CT images, particularly in low-dose scenarios, has the potential to improve the accuracy of automatic medical image segmentation further [19,20,21].

DEsCT was widely used in pediatrics, providing more diagnostic information, including tumors, lung perfusion, and metal implants [22,23,24,25]. Low-keV imaging reduced the usage of contrast agents in pediatric CT [26], enhanced the sensitivity in detecting children’s pulmonary embolism [27], and minimized the metal implant-based artifacts [28]. In the field of cardiology, low-keV imaging demonstrates the potential to reduce radiation doses and contrast agent usage while simultaneously offering the capability to differentiate materials with varying atomic numbers, which holds significant clinical implications [29,30]. Several powerful reconstruction algorithms adopted DEsCT to further improve the segmentation accuracy of radiotherapy images [31]. The iterative reconstruction algorithm improved clinical image segmentation results using model-based iterative reconstruction (MBIR) [32]. However, different VMI performances with DLIR on the low-density object’s segmentation accuracy, particularly in reduced radiation dose conditions, have not been comprehensively investigated. Although there is no significant statistical difference between DEsCT and single-energy CT, a series of slight differences, including CT attenuation, may potentially impact segmentation accuracy. Additionally, different energy levels and algorithms also influence the results [32,33,34]. Previously, segmentation methods demonstrated that deep learning reconstruction algorithms combined with spectral CT can reduce the radiation doses. Sixty keV is an effective value to maintain image quality while decreasing radiation exposure in pediatric CT [35]. Accurate segmentation plays a crucial role in measuring organ residual volume and informing clinical decisions [5,36,37].

The U-Net architecture has significantly advanced the domain of medical image segmentation by introducing an efficient and robust methodology for pixel-level image prediction [38,39]. A primary innovation of the architecture is its encoder-decoder structure, which facilitates the model’s ability to capture both high-level semantic features and detailed spatial information. The incorporation of skip connections between corresponding encoder and decoder layers ensures the preservation of high-resolution details, which is essential for tasks such as segmenting small or complex structures in medical images. This architecture has demonstrated high efficacy in applications such as tumor detection, organ segmentation, and other medical imaging tasks, yielding accurate segmentation results even with limited training data. The design of U-Net also supports training on smaller datasets, rendering it particularly valuable in the medical field, where annotated data are often scarce and costly to obtain. Its capability to produce precise segmentation maps has substantially enhanced the analysis and diagnosis of medical conditions from imaging data.

In this study, a low-resolution, non-enhancing object was used, and a U-Net deep learning algorithm was employed for automatic segmentation. The accuracies of the DLIR algorithm of low (DLIR-L), medium (DLIR-M), and high (DLIR-H) strength levels in scans with 50% of the standard radiation dose were compared with those of traditional CT reconstruction algorithms (filtered back-projection: FBP and ASIR-V) in segmenting low-density lesions. This method is derived from pediatric clinical application. When the radiation dose is reduced by half, segmentation tasks become more challenging, as noted in some studies examining model accuracy in the low-dose domain [40]. Utilizing phantom models addresses the ethical concerns associated with scanning pediatric patients [41]. Moreover, deep learning segmentation models for pediatric applications require parameter adjustments relative to adult models to achieve optimal performance [42]. The VMIs of various energy levels in DEsCT were also introduced to study the impact of different energy levels on segmentation accuracy. This approach highlights the potential of integrating DEsCT with DLIR to enhance the precision and effectiveness of medical imaging in clinical settings [43,44,45].

## 2. Materials and Methods

### 2.1. Phantom

**Figure 1:**
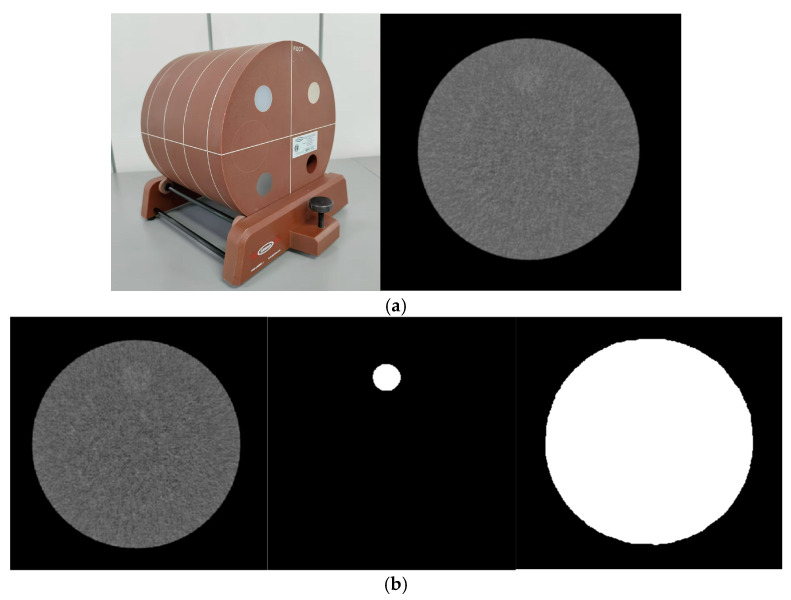
(**a**) The scan image of phantom, (**b**) The gold standard region of interest (ROI) of image and mask for low-density object, respectively.

This study involved phantom experiments that did not require ethical approval. An ACR 464 phantom (Phantom Laboratory Inc., Gammex, WI, USA) was employed in this study, as depicted in Figure 1a. Module 2, with an object of 6HU in density difference (DCT) and 25 mm in diameter, was utilized in this study. And Raw, ROI, and Maskmask were shown in Figure 1b.

### 2.2. CT Systems, Parameters for Acquisition and Reconstruction

All scans were performed on a 256-row CT (Revolution Apex, GE Healthcare, Milwaukee, WI, USA) utilizing the DEsCT imaging mode of fast tube voltage switching at two different dosage levels (volume CT dose indexes: CTDIvol of 5 mGy and 10 mGy determined on a 32 cm diameter reference phantom). For the 10 mGy scan, the tube current was set to 405 mA with a pitch of 0.992:1. For the 5 mGy scan, the tube current was set to 280 mA with a pitch of 1.375:1 [46]. The device was operated at a rotation speed of 0.5 s, captured images were captured with a pixel matrix size of 512 × 512. All the reconstructions were performed using a standard kernel with a slice thickness of 0.625 mm. The detector width along the z-axis was 40 mm. Six sets of virtual monochromatic images (VMIs) at energy levels of 40, 50, 60, 68, 74, and 100 keV were obtained. For the 10 mGy data acquisition, images were reconstructed using the standard ASIR-V at 50% strength level (ASIR-V50%), the currently commonly used reconstruction in clinical practice, and the default reconstruction weighting factor used in our center. For the 5 mGy data acquisition, images were reconstructed using the following algorithms: FBP, ASIR-V50% and 100% (ASIR-V100%) strength, and DLIR at low (DLIR-L), medium (DLIR-M), and high (DLIR-H) settings.

### 2.3. Deep Learning Model Construction

Model Training: The U-Net model training involved patching, which entailed cropping the original images into smaller sections containing low-density regions to augment the dataset for segmentation model training. The gold standard region of interest (ROI) and mask for low-density objects were delineated by two radiologists with ten years and three years of experience in radiology. Prior to the formal outlining process, all participating radiologists underwent standardized training, which included the use of consistent image display settings (e.g., window width and position) and outlining tools. The gold standard segmentation images were manually drawn to highlight regions with a slightly higher density. Masks were carefully outlined within the circular regions of the phantom, excluding the phantom frame and bed, to prevent interference in segmentation. As shown in Figure 1b. The original images were used for segmentation in the final test. A lower radiation dose was applied to amplify minor differences between the algorithms, helping to establish the applicable range and accuracy of each algorithm.

Training Set: The training set consisted of 10 mGy images reconstructed with ASIR-V50%, and the training set included eight images per energy level. In total, 48 images from 6 different VMIs were selected in this study; 80% of the images were utilized for model training, and 20% of the images were adopted for model evaluation.

Test Set: The 5 mGy images were reconstructed using the FBP, ASIR-V50%, ASIR-V100%, DLIR-L, DLIR-M, and DLIR-H algorithms across energy levels of 40, 50, 60, 68, 74, and 100 keV, resulting in 36 sets of images. Each set includes three consecutive slices of an object, with 108 sets of images in total. Four diagnostic doctors determined the optimal window width and window level for each energy level based on their clinical experience, yielding 12 segmentation results per set.

**Figure 2:**
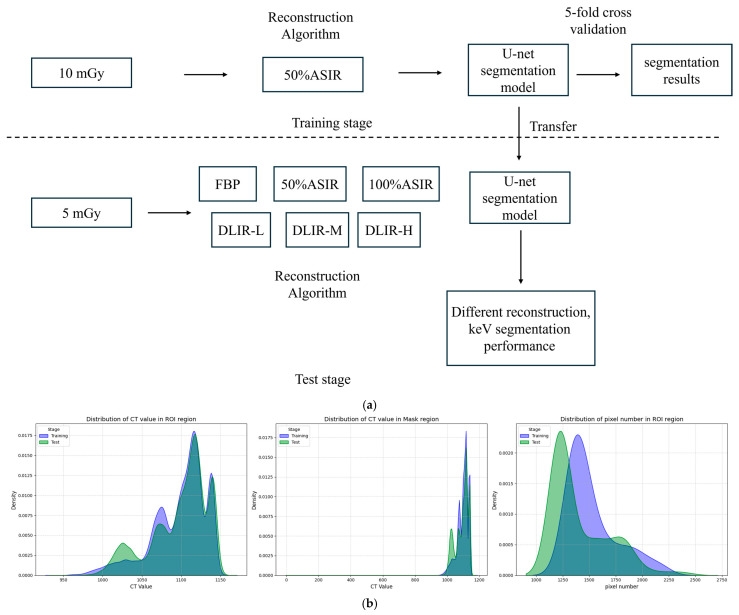
(**a**) The pipeline of our performance evaluation method. (**b**) The distribution of the dataset. Note: (**b**) shows the Kernel Density Estimation (KDE) curves of the training set and test set. The left figure represents the distribution of CT value in the ROI region, the middle figure represents the distribution of CT value in the mask region, and the right figure represents the pixel number in the ROI region.

The pipeline of our performance evaluation method and the distribution of the dataset are shown in Figure 2.

### 2.4. Metrics for Deep Learning Automatic Segmentation Evaluation

To evaluate the performance of the deep learning automatic segmentation, several metrics were used, including the Jaccard Index (intersection over union, IOU), DICE Coefficient (DICE), sensitivity, and Hausdorff distance (consisting of Manhattan, Euclidean, and Cosine distances). The calculation formula could be found in Appendix A.

### 2.5. Deep Learning Segmentation Model

To evaluate the performance of the deep learning automatic segmentation, several metrics were used, including the Jaccard Index (intersection over union, IOU), DICE Coefficient (DICE), sensitivity, and Hausdorff distance (consisting of Manhattan, Euclidean, and Cosine distances).

These metrics were utilized to assess the precision and effectiveness of the deep learning image reconstruction algorithm (TrueFidelityTM, GE Healthcare, Milwaukee, WI, USA) compared to traditional CT reconstruction algorithms for segmenting low-density lesions.

The deep learning segmentation model was developed using Python 3.8 and PyTorch 1.9.1. The results were visualized using the Matplotlib 3.6 library (NumFOCUS, Austin, TX, USA). The hardware setup included an Core i7-10700K (for USA) (Intel, Santa Clara, CA, USA) processor and an RTX 2080 Super graphics card (NVIDIA, CA, USA). The specific parameters of this study are as follows.

We developed a convolutional neural network based on the U-Net architecture for CT image segmentation tasks. The model mainly consists of an encoder and a decoder. The encoder part included an adaptation layer and four consecutive down-sampling layers. The adaptation layer is a convolutional module used to adapt to different channel inputs, with input and output channels of 1 and 64 channels, respectively. The down-sampling layers consist of convolutional, max-pooling, and activation modules. The convolutional modules output 64, 128, 256, and 512 channels, respectively, for local feature extraction without changing the feature dimensions. The max-pooling layers have a window size of two to extract the most distinctive features. The activation layer uses the ReLU to provide nonlinearity to the model. Stacking multiple down-sampling layers increases the depth of the model and effectively extracts global features from the CT images for subsequent segmentation tasks.

The decoder consists of four consecutive up-sampling layers. Bilinear interpolation layers were used to decode the global features and restore the original image size. The skip connection mechanism effectively mitigates gradient dispersion and accelerates model convergence; therefore, we symmetrically incorporated convolutional modules identical to those in the down-sampling layers into the up-sampling layers and skip connections of the output features. The final convolutional layer with two channels is used for foreground–background classification at each pixel, providing the segmentation results.

All trainable parameters were randomly initialized in a standard normal distribution and optimized using standard backpropagation with stochastic gradient descent by minimizing the loss of cross-entropy. The loss can be formulated as follows:

$$L_{ce}=\frac{1}{N}\sum_{i}L_{i}=-\frac{1}{N}\sum_{i}\sum_{M}^{c}y_{ic}\log(p_{ic})$$

where c is the total number of classes (in this study, foreground and background). $y_{ic}$ is the one-hot encoding of the pixel’s actual label. For class i, $y_{ic}$ = 1 indicates that the pixel belongs to class i; otherwise, $y_{ic}$ = 0, $p_{ic}$ is the probability of the pixel belonging to class i as predicted by the model. The Adadelta gradient descent algorithm (Adam) [47,48] with a batch size of 4 was used to train our model. Integrated L2 regularization (weight decay coefficient 0.0001) in Adam was used to stabilize training.

### 2.6. Measurement of CT Attenuation and Noise (Standard Deviation, SD)

CT attenuation and image noise of the low-contrast resolution phantom were gauged using ImageJ 1.54 (National Institutes of Health, Bethesda, MD, USA). ImageJ is an open-source image processing and analysis software widely used in scientific research for visualizing, quantifying (such as length, area, and pixel intensity), and segmenting images, with a modular architecture that supports user-defined plugin extensions for multidisciplinary applications [49]. The CT values and standard deviations (SD) were obtained by positioning a circular region of interest (ROI) with an area equal to half of the low-density resolution insert and a layer thickness of 5 mm. Subsequently, a comparably sized circular ROI was placed in the background to measure the CT and SD values. Measurements were taken ten times, and the average values were recorded. A subjective evaluation of medical images was conducted by three physicians who reviewed the images and provided a subjective quality assessment of the VMIs.

## 3. Results

**Figure 3:**
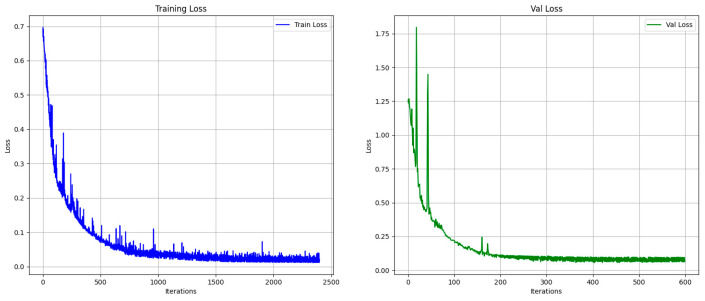
Training loss and Val loss.

**Figure 4:**
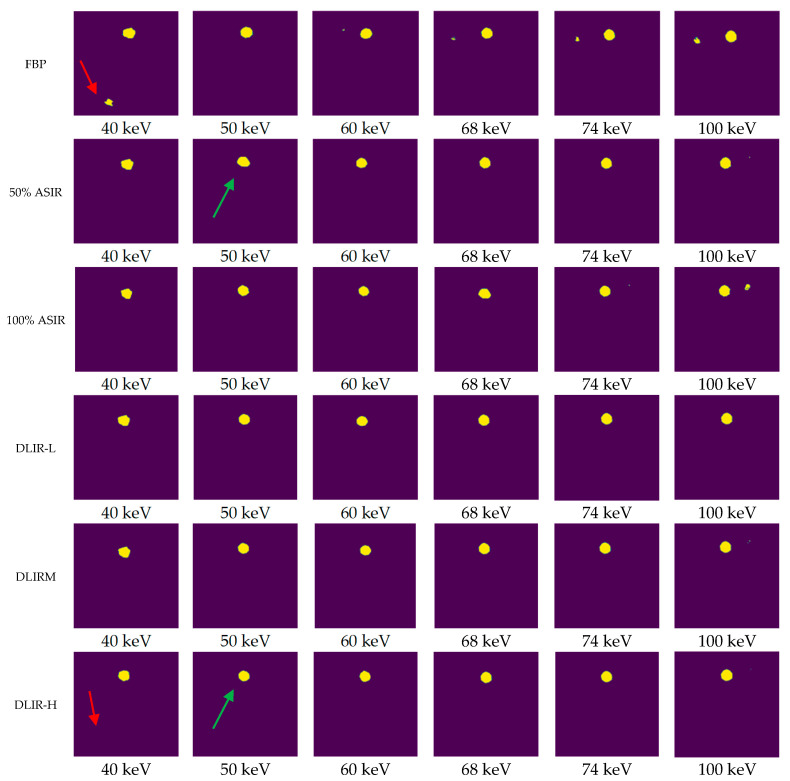
Segmentation results of low contrast resolution (1 case). Note: The areas indicated by the red arrows indicate that the phantom processed using the FBP algorithm exhibited numerous erroneous segmentation points in the lower-left corner. After processing with the DLIR-H algorithm, segmentation errors were eliminated. The positions indicated by the green arrows demonstrate that the ROI circle of the phantom was highly irregular when the 50% ASIR algorithm was used. In contrast, after processing using the DLIR-H algorithm, the ROI circle became more regular. The displayed segmentation results of the phantom reflect the actual segmentation performance more intuitively.

The segmentation model achieved a reliable training performance, with an accuracy of 99.6% on the training set. The loss curves are shown in Figure 3. The segmentation results with different algorithms (VMls) and photon energy levels are shown in Figure 4.

### 3.1. Metrics for Deep Learning Automatic Segmentation in Validation Set (5 mGy)

#### 3.1.1. Performance Metrics (IOU, DICE, and Sensitivity)

The specific IOU values as a function of the energy levels for FBP, ASIR-V50%, ASIR-V100%, DLIR-L, DLIR-M, and DLIR-H are as follows: 60 keV: 0.60, 0.67, 0.68, 0.72, 0.75, and 0.75; 68 keV: 0.62, 0.67, 0.70, 0.71, 0.72, and 0.72; 74 keV: 0.62, 0.65, 0.67, 0.69, 0.69, and 0.69, respectively. The specific DICE values for FBP, ASIR-V50%, ASIR-V100%, DLIR-L, DLIR-M, and DLIR-H were as follows: 60 keV: 0.75, 0.80, 0.82, 0.83, 0.85 and 0.86; 68 keV: 0.76, 0.80, 0.80, 0.83, 0.84 and 0.83; 74 keV: 0.75, 0.78, 0.80, 0.81, 0.81 and 0.81, respectively. The specific sensitivity values for FBP, ASIR-V50%, ASIR-V100%, DLIR-L, DLIR-M, and DLIR-H were as follows: 60 keV: 0.93, 0.91, 0.96, 0.95, 0.98, and 0.98; 68 keV: 0.84 0.85, 0.92, 0.93, 0.93, and 0.95; 74 keV: 0.83, 0.88, 0.93, 0.96, 0.97, and 0.98, respectively.

**Figure 5:**
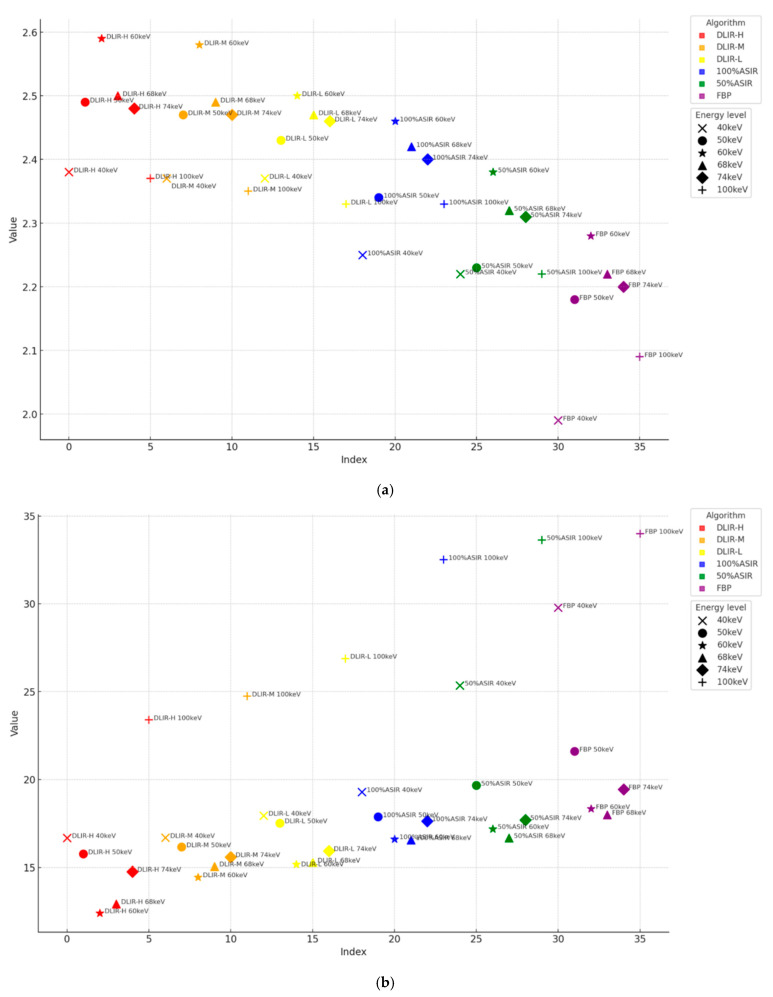
(**a**) The sum of IOU, DICE, and sensitivity in different algorithms at different energy levels. (**b**) The sum of the Hausdorff distance in different algorithms at different energy levels.

**Figure 6:**
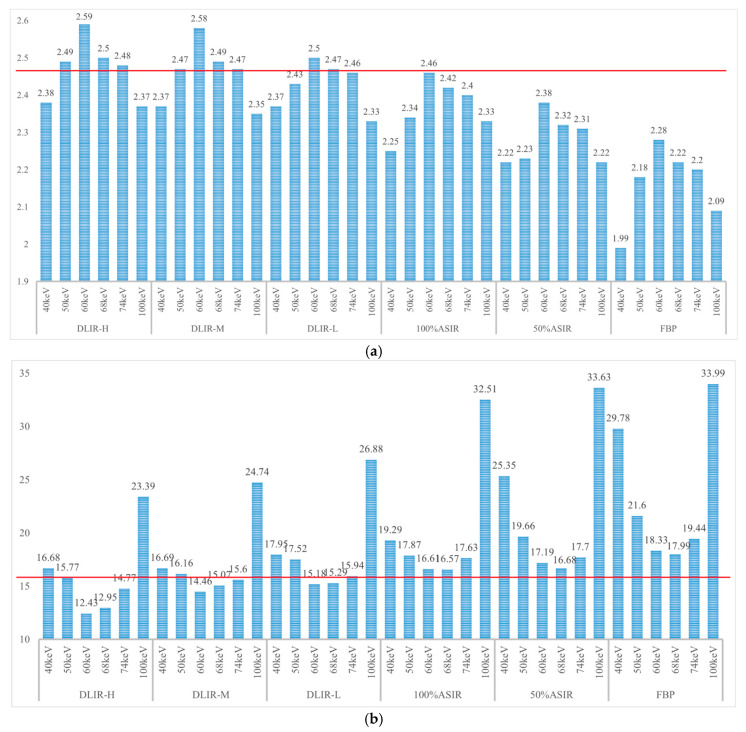
(**a**) The sum of IOU, DICE, and sensitivity in different algorithms at different energy levels. (**b**) The sum of the Hausdorff distance in different algorithms at different energy levels. Note: The DLIR-H reconstruction algorithm can significantly improve the segmentation accuracy at 50 keV, which is superior to all other Asir-V algorithms and keV, making it possible to further reduce the amount of contrast agent.

With energy levels (keV), the performance metrics (IOU, DICE, and sensitivity) ranked from the highest to the lowest as a function of the energy level for different reconstruction algorithms were, for the FBP and 100% ASIR-V algorithms, 60 keV, 68 keV, 74 keV, 50 keV, 100 keV, and 40 keV; for DLIR-H, DLIR-M, DLIR-L, and 50% ASIR-V, they were 60 keV, 68 keV, 74 keV, 50 keV, 40 keV, and 100 keV. For reconstruction algorithms, the performance metrics were ranked from the highest to the lowest for different reconstruction algorithms, and over all energy levels were DLIR-H, DLIR-M, DLIR-L, 100% ASIR-V, 50% ASIR-V, and FBP. All the sum performance metrics results are shown in Figure 5a and Figure 6a. Detailed values are listed in the Appendix B (Table A1).

#### 3.1.2. Hausdorff Distance

The specific cosine distance values for FBP, ASIR-V50%, ASIR-V100%, DLIR-L, DLIR-M, and DLIR-H were as follows: 60 keV: 0.52, 0.49, 0.52, 0.51, 0.35, and 0.34; 68 keV: 0.48, 0.49, 0.58, 0.52, 0.43, and 0.42; 74 keV: 0.57, 0.54, 0.57, 0.41, 0.44, and 0.38. The specific Manhattan_distance values for FBP, ASIR-V50%, ASIR-V100%, DLIR-L, DLIR-M, and DLIR-H were as follows: 60 keV: 14.08, 13.17, 12.58, 11.42, 10.92, and 9.08; 68 keV: 13.83, 12.92, 12.50, 11.35, 11.31, and 9.5; 74 keV: 15.08, 13.52, 13.53, 12.08, 11.76, and 11.08. The specific Euclidean_distance values for FBP, ASIR-V50%, ASIR-V100%, DLIR-L, DLIR-M, and DLIR-H were as follows: 60 keV: 3.73, 3.53, 3.51, 3.25, 3.19, and 3.01; 68 keV: 3.68, 3.27, 3.49, 3.42, 3.33, and 3.03; 74 keV: 3.79, 3.64, 3.53, 3.45, 3.40, and 3.31.

With respect to energy levels: The Hausdorff distance ranked from the lowest to the highest as a function of energy level for different reconstruction algorithms were as follows: for FBP, 50% ASIR-V, and 100% ASIR-V algorithms, they were 68 keV, 60 keV, 74 keV, 50 keV, 40 keV, and 100 keV; for DLIR-H, DLIR-M, and DLIR-L, they were 60 keV, 68 keV, 74 keV, 50 keV, 40 keV, and 100 keV. Regarding the reconstruction algorithms, the Hausdorff distance ranked from the lowest to the highest for different reconstruction algorithms over all energy levels were DLIR-H, DLIR-M, DLIR-L, ASIR 100%, 50% ASIR, and FBP. All the Hausdorff results are shown in Figure 5b and Figure 6b. Detailed results are listed in the Appendix B (Table A1).

### 3.2. CT Attenuation and Standard Deviations (SDs) of the Dual-Energy Spectral CT Image

**Figure A1:**
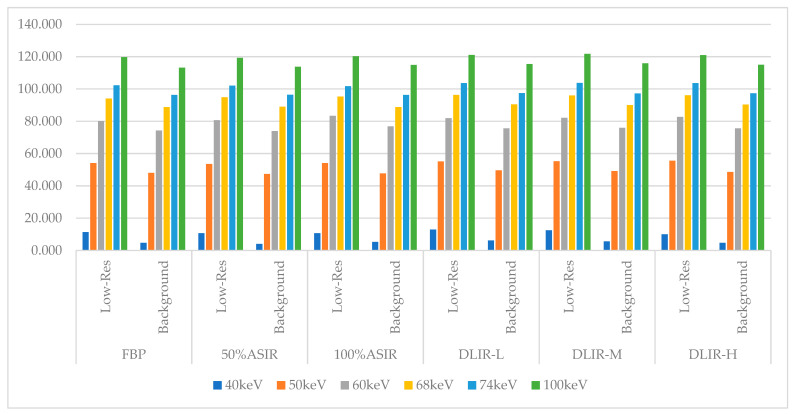
CT attenuation of the low-resolution phantom and background under different algorithms.

**Figure A2:**
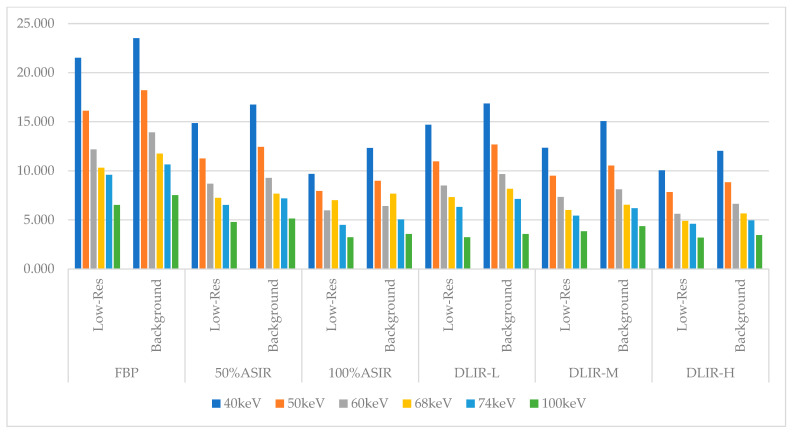
Image noise of the low-resolution phantom and background under different algorithms.

**Figure A3:**
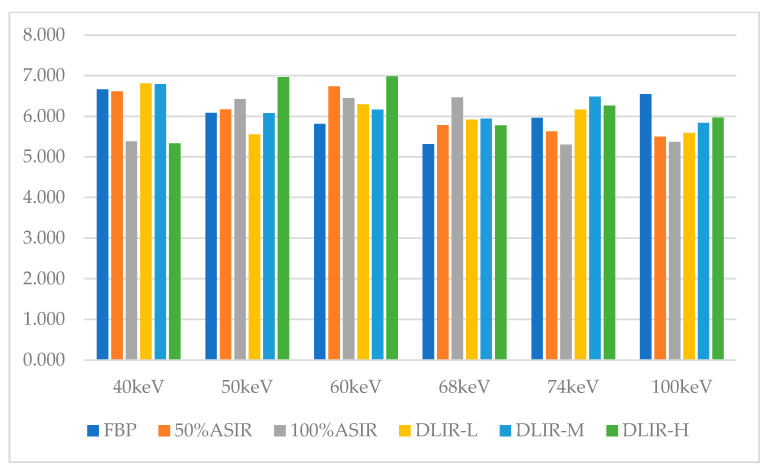
The difference between the low-resolution phantom and the background. Note: Low-res, low-resolution insert. Back-ground, back-ground of the insert.

The CT attenuation value changed with the energy level. At each energy level, the CT attenuation values were statistically the same across all reconstruction algorithms; however, the image noise values (SD) showed statistically significant differences. Table 1 shows the CT attenuation values for the low-resolution phantom and background under different algorithms, and Table 2 details the corresponding image noise levels. Table 2 demonstrates that as the keV increases, the standard deviation (SD) values of the low-density phantom in the images continuously decrease. The DLIR-H algorithm exhibits the lowest standard deviation (SD) value among all the algorithms. The differences observed in these data are statistically significant (*p* < 0.05). Table 3 shows the D-values between the low-resolution phantom and the background. Table 4 presents the subjective evaluation results of the different monochromatic images (validation sets) with different reconstruction algorithms. Specific charts are displayed in the Appendix C (Figure A1, Figure A2 and Figure A3).

As the energy level (keV) increased, the CT attenuation for the ASIR-V50% low-contrast resolution insert gradually increased with attenuation values of 10.70, 53.59, 80.73, 94.85, 102.03, and 119.28. In contrast, the noise levels decreased, with values of 14.86, 11.26, 8.68, 7.23, 6.52, and 4.78, respectively. Similarly, the CT attenuation of the background also increased, with values of 4.09, 47.42, 73.99, 89.07, 96.41, and 113.78, whereas the noise levels decreased to 16.75, 12.44, 9.27, 7.66, 7.19, and 5.14, respectively. The differences between the low-resolution insert and background across different energy levels (keV) and the reconstruction algorithms were not statistically significant. At each energy level, the DLIR-H provided the strongest ability to reduce image noise.

The specific IOU, DICE, sensitive average value was presented in Appendix B, Table A1. The average value of all the segmentation metrics is also provided.

Finally, the average results for the performance metric, Hausdorff distance, and image quality measurements for the reconstruction algorithms across all energy levels are listed in Table 5.

## 4. Discussion

This study comprehensively discussed the impact of different energy levels and different reconstruction algorithms in low-dose (5 mGy) DEsCT on the accuracy of automatic segmentation of low-density objects using an objective in DICEs. DEsCT is increasingly being adopted in clinical practice for imaging pediatric patients [22,23,50,51]. Integrating low-dose DEsCT imaging with deep-learning image reconstruction algorithms is a fundamental approach in clinical imaging, allowing the identification of the most effective combinations of reconstruction algorithms and energy levels [52]. Research involving pediatric phantoms has shown that low-dose, high-end CT devices can effectively guide ear surgery [53]. Our findings suggest that by using phantom experiments and objective indicators, deep learning image reconstruction algorithms can significantly reduce image noise and improve image quality in low radiation dose DEsCT for pediatric patients. Children are not simply smaller versions of adults. Different radiation doses and distinct histopathological features require consideration, and phantom model research will further guide pediatric clinical practice [42]. According to the literature, the optimal single energy level is 65 keV for the gray matter–white matter boundary in the brain, which aligns with the soft tissue boundary findings in our study, where 60 keV was found to generate the best segmentation accuracy for low-density, non-enhancing objects [54].

The accurate segmentation of lesions is crucial in clinical practice, particularly in pediatric patients. In the surgical evaluation of hepatoblastoma, precise segmentation techniques are essential to accurately delineate tumor boundaries, enabling a more accurate assessment of the extent of the tumor and its anatomical relationships with adjacent structures. Additionally, the postoperative evaluation of residual liver tissue was significantly improved using these segmentation methods. By accurately measuring the volume and distribution of the remaining liver parenchyma, clinicians can better predict a patient’s recovery trajectory and potential complications, ultimately contributing to more informed surgical planning and improved patient outcomes.

Our comprehensive evaluation highlights the effectiveness of integrating low-dose dual-energy spectral CT imaging with deep learning image reconstruction algorithms. The results demonstrated significant improvements in segmentation accuracy and image quality, particularly with the DLIR-M and DLIR-H algorithms, across all energy levels. These findings underscore the potential of advanced techniques to enhance clinical imaging and diagnostic precision.

We averaged the values across all six energy levels for the different reconstruction algorithms to evaluate their overall performance. The results indicated that DLIR-M and DLIR-H had similar values in IOU and DICE, which focused on internal filling and clinical indicators of the phantom and were among the best among all the reconstruction algorithms. We believe this is because of their ability to provide low image noise while maintaining image textures to achieve the most balanced segmentation and to correctly identify the majority of the segmented regions. In contrast, DLIR-H performed better in terms of sensitivity and the Hausdorff distance metric, which emphasizes the number and distance of misclassified points in the segmentation results, indicating that it had fewer segmentation errors.

DLIR-H provided images with significantly less noise than did DLIR-M. However, according to earlier research by Greffier, J, while DLIR-M noise reduction is not as effective as DLIR-H, the noise power spectrum (NPS) and image texture of DLIR-M are more similar to those of FBP [55]. In the field of image segmentation, the effectiveness of segmentation is closely related to both the image noise and texture. This also explains why, despite the much lower noise levels in ASIR-V100%, its segmentation performance was inferior to that of the DLIR-L series.

Low-energy images in dEsCT, such as 40 keV images, are most effective in imaging objects enhanced with iodine because of the closer proximity in energy value of 40 keV photons to the k-edge of iodine. However, in our study, a non-enhancing object simulating low-density lesions was used in clinical applications. From the measured CT attenuation, the poor segmentation accuracy at 40 keV can be attributed to the small differences between the low-contrast object and background. In our study, the contrast of the low-density object was not improved by using the 40 keV photons, whereas the image noise was significantly increased compared with that of the 60 keV images. In addition, the CT values of the object itself at 40 keV were significantly different from those of the 68 keV images on which the segmentation model was trained. All of these factors make lesion segmentation challenging. Similarly, at 100 keV, the significant changes in the image texture (image noise and CT value) likely contributed to the reduced segmentation accuracy. The potential value of this study lies in its ability to guide clinical practice. For example, the application of DLIR-H can enhance segmentation accuracy when targeting liver and other soft tissue lesions with varying densities. Additionally, the use of low-dose energy-spectrum CT, particularly at 60 keV, is more effective for pediatric cases. Moreover, body-modeling studies can help avoid ethical concerns. We have initiated trials of the model with clinical data, and we plan to report the results of this phase in future studies.

This study had several limitations. First, the data were collected from a single center using a specific low-resolution phantom, which did not represent all clinical scenarios, particularly contrast-enhanced lesions. It is necessary to expand the dataset to validate the robustness of the model further. Additionally, a specific ACR464 phantom was used in this study only to assess the success rate of automatic segmentation under the same CNR conditions without considering the extent to which the radiation dose and contrast agent usage could be reduced. Based on previous research, 50 keV has been shown to reduce both the radiation dose and the contrast agent usage more effectively. Finally, this study focused only on non-enhancing, low-density objects, without evaluating segmentation performance in the context of contrast-enhanced CT in actual clinical settings. Further validation is required, either with contrast phantoms containing different iodine concentrations or through clinical experiments.

## 5. Conclusions

In conclusion, a comprehensive analysis suggests that the combination of 60 keV and deep learning image reconstruction algorithms with medium or high strengths (DLIR-M and DLIR-H) delivers the best results for segmenting low-density objects, whereas DLIR-H provides images with the highest sensitivity and lowest image noise across all energy levels in dEsCT. In future medical applications, we will further fine-tune model parameters and test the applicability of applying DLIR-H in combination with 60 keV to improve the segmentation accuracy.

## Figures and Tables

**Table 1:** CT attenuation of the low-resolution phantom and background under different algorithms.

Recon	FBP		50% ASIR		100% ASIR		DLIR-L		DLIR-M		DLIR-H	
	Low-Res	Back-Ground	Low-Res	Back-Ground	Low-Res	Back-Ground	Low-Res	Back-Ground	Low-Res	Back-Ground	Low-Res	Back-Ground
40 keV	11.39	4.72	10.70	4.09	10.68	5.30	12.98	6.17	12.47	5.68	10.08	4.75
50 keV	54.18	48.10	53.59	47.42	54.10	47.68	55.20	49.64	55.31	49.23	55.65	48.68
60 keV	80.10	74.29	80.73	73.99	83.37	76.93	81.90	75.61	82.18	76.02	82.67	75.69
68 keV	94.10	88.78	94.85	89.07	95.27	88.81	96.39	90.47	95.97	90.03	96.13	90.35
74 keV	102.28	96.32	102.03	96.41	101.68	96.38	103.63	97.46	103.75	97.26	103.59	97.33
100 keV	119.80	113.25	119.28	113.78	120.24	114.87	121.06	115.47	121.74	115.90	120.96	114.99

**Table 2:** Standard deviations (SDs) of the low-resolution phantom and background under different algorithms.

Recon	FBP		50% ASIR		100% ASIR		DLIR-L		DLIR-M		DLIR-H	
	Low-Res	Back-Ground	Low-Res	Back-Ground	Low-Res	Back-Ground	Low-Res	Back-Ground	Low-Res	Back-Ground	Low-Res	Back-Ground
40 keV	21.51	23.51	14.86	16.75	9.68	12.32	14.70	16.87	12.34	15.07	10.06	12.04
50 keV	16.12	18.20	11.26	12.44	7.94	8.98	10.95	12.67	9.50	10.53	7.84	8.84
60 keV	12.18	13.91	8.675	9.27	7.00	6.41	8.49	9.67	7.34	8.12	5.61	6.64
68 keV	10.31	11.75	7.23	7.66	5.96	7.67	7.32	8.17	6.00	6.53	4.89	5.66
74 keV	9.59	10.65	6.52	7.19	4.48	5.04	6.31	7.14	5.43	6.18	4.60	4.95
100 keV	6.51	7.53	4.78	5.14	3.22	3.55	4.60	3.55	3.83	4.35	3.19	3.45

**Table 3:** Difference (D-value) of the low-resolution phantom and background.

Recon	FBP	50% ASIR	100% ASIR	DLIR-L	DLIR-M	DLIR-H
40 keV	6.67	6.61	5.38	6.81	6.79	5.33
50 keV	6.09	6.17	6.42	5.56	6.08	6.97
60 keV	5.81	6.74	6.45	6.29	6.17	6.99
68 keV	5.31	5.78	6.47	5.92	5.94	5.78
74 keV	5.96	5.63	5.30	6.16	6.48	6.26
100 keV	6.55	5.50	5.37	5.59	5.84	5.97

**Table 4:** Subjective evaluation results of images in the validation group at different energy levels and with different reconstruction algorithms.

Recon	FBP	50% ASIR	100% ASIR	DLIR-L	DLIR-M	DLIR-H
40 keV	1.00	2.00	1.67	2.00	2.00	2.33
50 keV	1.50	2.50	2.33	3.00	3.33	3.33
60 keV	2.00	3.00	2.67	3.00	3.33	3.33
68 keV	2.00	3.00	2.67	3.00	3.33	3.33
74 keV	2.00	3.00	3.00	3.00	3.33	3.67
100 keV	1.67	2.00	2.00	2.33	2.67	3.00

**Table 5:** Average values (over six energy levels) of metrics related to the segmentation results (ALL 40–100 keV).

Recon	IOU	DICE	Sensitivity	Manhattan Distance	Euclidean Distance	Cosine Distance	BKG Noise	Quality Score
FBP	0.58	0.72	0.86	18.64	4.26	0.63	14.26	1.70
50% ASIR	0.62	0.76	0.90	17.11	4.00	0.60	9.74	2.58
100% ASIR	0.65	0.78	0.93	15.66	3.85	0.57	7.33	2.39
DLIR-L	0.67	0.80	0.95	13.93	3.67	0.53	10.01	2.72
DLIR-M	0.68	0.81	0.96	13.10	3.55	0.48	8.46	3.00
DLIR-H	0.69	0.81	0.97	12.14	3.42	0.44	6.93	3.17

Note: IOU, intersection over union. DICE, DICE similarity coefficient. Hausdorff distance includes Manhattan_distance, Euclidean_distance, and Cosine_distance.

## Data Availability

The data presented in this study are available on request from the corresponding author.

## Abbreviations

The following abbreviations are used in this manuscript:

ASIR-V	Adaptive statistical iterative reconstruction-veo
CTDIvol	CT dose index
DEsCT	Dual-energy spectral computed tomography
DICE	DICE coefficient
DLIR	Deep learning image reconstruction
FBP	Filtered back-projection
HU	Hounsfield Unit
IOU	Intersection over union
IR	Iterative reconstruction
keV	Kilo-electron volts
NPS	Noise power spectrum
ROI	Region of interest
SD	Standard deviation
U-Net	Convolutional Networks for Biomedical Image Segmentation
VMIs	Virtual monochromatic images

## Appendix A. Evaluation Index of the Accuracy of Deep Learning Segmentation Model

Jaccard Index (intersection over union, IOU):

The Jaccard Index is a statistical measure that compares the similarity and diversity between two sets. It is defined as the ratio of the size of the intersection of the two sets to the size of their union. In this study, the Jaccard Index is primarily used to evaluate how closely the segmentation results from the U-Net model align with the gold standard delineated by physicians. An IOU value closer to 1 indicates a higher degree of similarity. The formula for IOU is as follows:

$$IOU=\frac{A\cap B}{A\cup B}=\frac{TP}{FP+TP+FN}$$

The DICE coefficient is a metric used to measure the overlap between two sets, similar to the Jaccard Index but giving more weight to the size of the overlapping area. It is particularly useful in segmentation tasks where precise overlap is crucial. The formula for the DICE coefficient is as follows:

$$Dice=\frac{2\left|A\cap B\right|}{\left|A\right|+\left|B\right|}=\frac{2TP}{FP+2TP+FN}$$

Sensitivity, also known as recall, measures the proportion of actual positives that the model correctly identifies. The formula for sensitivity is as follows:

$$Sensitivity=\frac{TP}{TP+FN}$$

PPV, also known as precision, measures the proportion of positive results that are true positives. The formula for PPV is as follows:

$$PPV=\frac{TP}{TP+FP}$$

In these metrics: True Positive (TP): The model predicts a positive sample, and the actual result is also a positive sample. False Positive (FP): The model predicts a positive sample, but the actual result is a negative sample. True Negative (TN): The model predicts a negative sample, and the actual result is also a negative sample. False Negative (FN): The model predicts a negative sample, but the actual result is a positive sample.

The Hausdorff distance measures the degree of resemblance between two sets of points. It calculates the maximum distance from a point in one set to the nearest point in the other set. Variants include the following:

$$d_{H}\left(X,Y\right)=max\left\{\underset{\mathrm{x\epsilon X}}{\max}\underset{\mathrm{y\epsilon Y}}{\min}d\left(x,y\right),\underset{\mathrm{y\epsilon Y}}{\max}\underset{\mathrm{x\epsilon X}}{\min}d\left(x,y\right)\right\}$$

Manhattan distance: The sum of the absolute differences between the Cartesian coordinates of two points.

Euclidean distance: The straight-line distance between two points in Euclidean space.

Cosine distance: Measures the cosine of the angle between two vectors.

## Appendix B

**Table A1:** The average value of the metrics related to the segmentation results (All).

		IOU	DICE	Sensitivity	Manhattan_ Distance	Euclidean_ Distance	Cosine_ Distance
40 keV	FBP	0.48	0.63	0.88	24.17	4.84	0.77
	50% ASIR	0.57	0.72	0.93	20.25	4.42	0.68
	100% ASIR	0.59	0.74	0.92	14.92	3.82	0.55
	DLIR-L	0.63	0.77	0.97	13.75	3.69	0.51
	DLIR-M	0.61	0.77	0.99	12.67	3.53	0.49
	DLIR-H	0.63	0.76	0.99	12.83	3.46	0.39
50 keV	FBP	0.59	0.74	0.85	16.92	4.07	0.61
	50% ASIR	0.62	0.76	0.85	15.25	3.85	0.56
	100% ASIR	0.65	0.78	0.91	13.75	3.70	0.42
	DLIR-L	0.69	0.81	0.93	13.42	3.64	0.46
	DLIR-M	0.7	0.83	0.94	12.25	3.49	0.42
	DLIR-H	0.72	0.82	0.95	11.92	3.42	0.43
60 keV	FBP	0.60	0.75	0.93	14.08	3.73	0.52
	50% ASIR	0.67	0.80	0.91	13.17	3.53	0.49
	100% ASIR	0.68	0.82	0.96	12.58	3.51	0.52
	DLIR-L	0.72	0.83	0.95	11.42	3.25	0.51
	DLIR-M	0.75	0.85	0.98	10.92	3.19	0.35
	DLIR-H	0.75	0.86	0.98	9.08	3.01	0.34
68 keV	FBP	0.62	0.76	0.84	13.83	3.68	0.48
	50% ASIR	0.67	0.80	0.85	12.92	3.27	0.49
	100% ASIR	0.70	0.80	0.92	12.5	3.49	0.58
	DLIR-L	0.71	0.83	0.93	11.35	3.42	0.52
	DLIR-M	0.72	0.84	0.93	11.31	3.33	0.43
	DLIR-H	0.72	0.83	0.95	9.50	3.03	0.42
74 keV	FBP	0.62	0.75	0.83	15.08	3.79	0.57
	50% ASIR	0.65	0.78	0.88	13.52	3.64	0.54
	100% ASIR	0.67	0.80	0.93	13.53	3.53	0.57
	DLIR-L	0.69	0.81	0.96	12.08	3.45	0.41
	DLIR-M	0.69	0.81	0.97	11.76	3.40	0.44
	DLIR-H	0.69	0.81	0.98	11.08	3.31	0.38
100 keV	FBP	0.55	0.70	0.84	27.75	5.42	0.82
	50% ASIR	0.56	0.71	0.95	27.52	5.30	0.81
	100% ASIR	0.61	0.76	0.96	26.67	5.06	0.78
	DLIR-L	0.60	0.75	0.98	21.58	4.54	0.76
	DLIR-M	0.62	0.76	0.97	19.67	4.34	0.73
	DLIR-H	0.63	0.77	0.97	18.42	4.28	0.69
